# A New Single Nucleotide Polymorphism Database for North American Atlantic Salmon Generated Through Whole Genome Resequencing

**DOI:** 10.3389/fgene.2020.00085

**Published:** 2020-02-21

**Authors:** Guangtu Gao, Michael R. Pietrak, Gary S. Burr, Caird E. Rexroad, Brian C. Peterson, Yniv Palti

**Affiliations:** ^1^ National Center for Cool and Cold Water Aquaculture, ARS-USDA, Kearneysville, WV, United States; ^2^ National Cold Water Marine Aquaculture Center, ARS-USDA, Orono, ME, United States; ^3^ USDA ARS Office of National Programs, George Washington Carver Center, Washington, D.C., United States

**Keywords:** Atlantic salmon, SNP discovery, whole genome re-sequencing, aquaculture stocks, continent of origin, North American

## Introduction

The Atlantic salmon is the most economically important species of the family Salmonidae. As a group, salmonid species hold considerable economic, social, and environmental importance in Europe, the Americas, and Australia (Davidson et al., 2010). Compared to other economically important fish species and aquatic model research organism, the Atlantic salmon genome is very large. The genome size estimate for Atlantic salmon based on cellular DNA content is 3.0 Gbp (Hardie and Hebert, 2003). Although it is similar in size to that of most mammals, its architecture and organization are more complex. All ray-finned fish share an additional (3R) round of ancestral genome duplication in their evolutionary history compared to mammals and birds, but the salmonids underwent an additional recent (4R) whole genome duplication event (Allendorf and Thorgaard, 1984; Lien et al., 2016). In total, 94.4% of the Atlantic salmon genome is still composed of 98 pairs of colinear blocks (196 regions) of high sequence similarity known as homeologous chromosome regions. This includes approximately 25% of the genome assembly in seven pairs of chromosome arms with elevated sequence similarity (>90%) and patterns of tetrasomic inheritance (Lien et al., 2016). In addition, nearly 60% of the Atlantic salmon genome contain repetitive sequences (Lien et al., 2016).

Single-nucleotide polymorphisms (SNP) are highly abundant markers broadly distributed in animal genomes. High density SNP arrays are used for collecting large amount of genome-wide genotype data. This information is useful for dissecting the genetic basis of quantitative traits in agriculture and for implementing models of genomic selection, which has revolutionized the field of selective breeding over the past decade (Meuwissen et al., 2016). High density SNP arrays are publicly available for Atlantic salmon of European origin (Houston et al., 2014). However, Atlantic salmon farming in eastern US and Canada is restricted to genetic stocks of North American (NA) origin due to ecological and conservation concerns. The NA Atlantic salmon is a different sub-species with substantial genomic differences from the European sub-species (Lubieniecki et al., 2010; Brenna-Hansen et al., 2012). Approximately 50% of the SNPs from the arrays designed for the European salmon are informative for NA salmon (James Kijas, personal communication) (Yáñez et al., 2016). Other researchers have developed optimized SNP chips for salmon of NA origin based on experimental information from the larger European salmon arrays, but those are currently not available for the public due to intellectual property (IP) constrains from competitive commercial interests (Kijas et al., 2018).

Recently, we initiated efforts to generate genomic research resources for the breeding program at the USDA-ARS National Cold Water Marine Aquaculture Center (NCWMAC). We used high coverage whole genome Illumina resequencing for SNP discovery in 80 NA Atlantic salmon individuals from three aquaculture stocks that are propagated in the NCWMAC. Sequences of four doubled haploid (DH) European Atlantic salmon were added to the analysis to detect putative paralogous sequence variants and multi-sequence variants following the SNP discovery analysis methods we previously described (Gao et al., 2018). Overall, we discovered about 6.6 million SNP markers, including over 1.5 million markers having high minor allele frequency (MAF ≥ 0.25). In addition, we identified 5,822 candidate markers that can potentially distinguish between NA and European Atlantic salmon by comparing genotypes of the 80 NA Atlantic salmon with publicly available whole genome sequence information from 31 Atlantic salmon representing a diversity of European populations. The SNP database we generated from this work provides an important resource for a high-density SNP array design, as well as for other SNP genotyping platforms that can be used for genetic and genomics studies of NA Atlantic salmon.

## SNP Data

**Figure 1:**
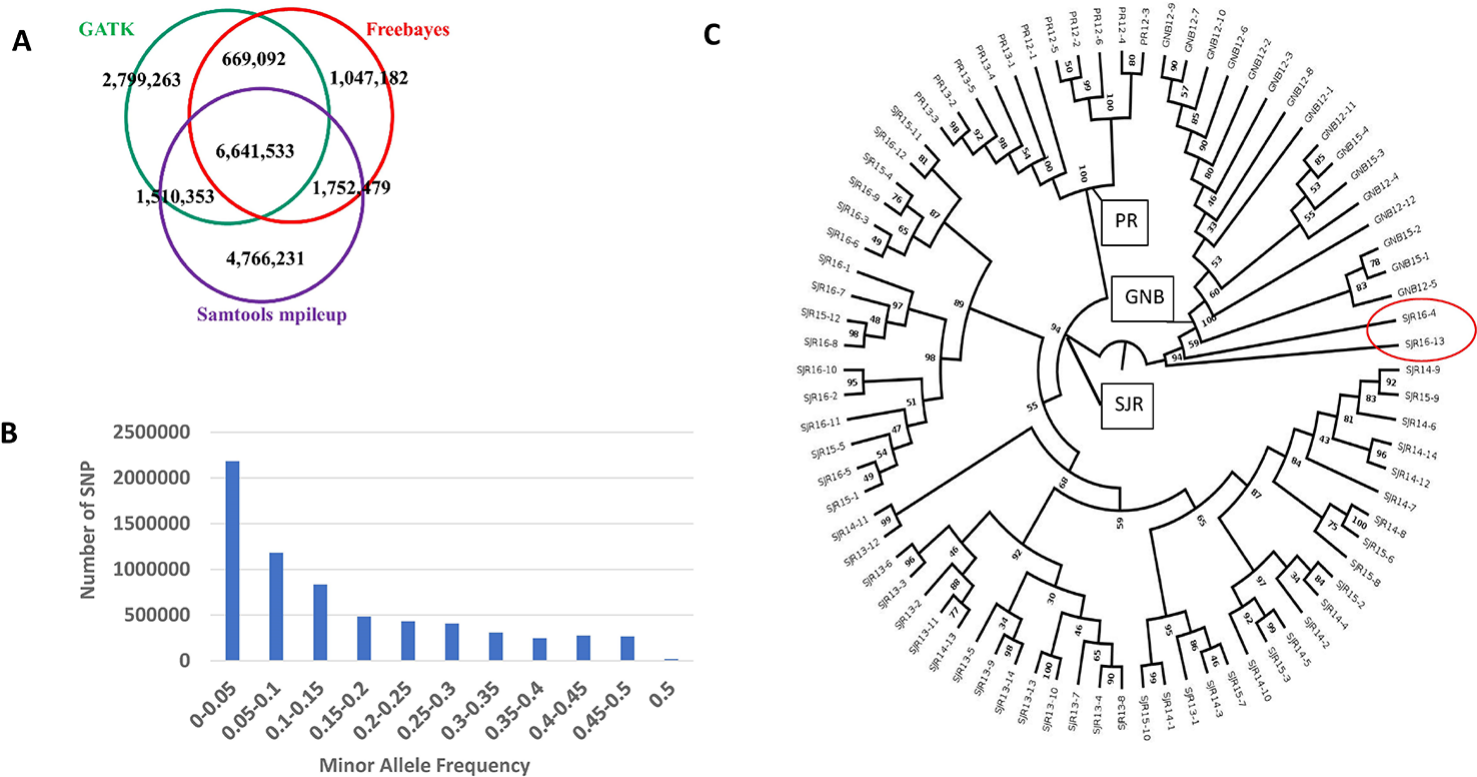
A summary of how many SNPs are shared among the three pipelines is displayed as a Venn diagram **(A)**. Distribution of minor allele frequency (MAF) among the 6,641,533 SNPs that are shared by all three pipelines **(B)**. A phylogenetic tree of the 80 North American Atlantic salmon sampled from three aquaculture strains and up to four year classes within a strain **(C)**. The number at each node represents the bootstrap value (percentage out of 1,000 bootstrap samples). The strain and first two digits of the year-class are represented in the sample name.

The number of SNPs we identified using the freebayes, mpileup, and GATK pipelines were 10,110,286, 14,670,596, and 11,620,241, respectively. More specific information about each SNP can be found online in the European Variation Archive (EVA) in the Variant Call format (vcf) (under project PRJEB34225; Accessions ERZ107004, ERZ107005, and ERZ107006). A summary of how many SNPs are shared among the three pipelines is displayed as a Venn diagram in **Figure 1A**. Overall, 6,641,533 SNPs were called by all three pipelines with genotypes from at least 79 NA Atlantic salmon. The distribution of minor allele frequency (MAF) among those 6,641,533 SNPs is shown in **Figure 1B** with 1,527,345 SNPs (23%) having high minor allele frequency (MAF ≥ 0.25). To assess the utility of the new SNP database for analyses of polymorphism between the three aquaculture strains we generated a phylogenetic tree (**Figure 1C**). Each strain was clearly separated to a different branch except for two ungrouped fish from the 16–17 year-class (YC) of the St. John River (SJR) strain (Fish IDs SJR 16-4 and SJR 16-13). Currently, we cannot exclude the possibility that some hybridization has occurred in a previous generation in the pedigree of those two fish between SJR fish and a fish from one of the two other aquaculture strains of NA origin, hence resulting in our inability to group them with any of the three distinct groups. Within the strain, the branches the fish were overall grouped according to YC. However, within the SJR strain the grouping of fish on three main branches reflected the practice of using parents from two adjacent YCs. One branch grouped fish from the 15–16 and the 16–17 YCs, a second branch grouped fish from the 13–14 and the 14–15 YCs and the third branch grouped fish from YCs 14–15 and 15–16. To assess marker polymorphism within each individual strain and YC, we counted the number of polymorphic markers and the heterozygosity of the individual markers in each strain and YC within strain (**Table 1**). The marker polymorphism was very high in the SJR strain with 95% of the SNPs being polymorphic in that strain and over 70% being polymorphic within each YC. It was lower in the Gaspe of New Brunswick (GNB) strain and the Penobscot River (PR) strain with 54% of the SNPs being polymorphic within each strain and between 39 and 47% within each YC. At the same time, the average marker heterozygosity among the SNPs that were polymorphic in each strain was highest in the GNB strain (47%), followed by the PR strain (35%) and then the SJR (23%). This lower polymorphism and higher heterozygosity of the markers that are polymorphic in the GNB and PR strains reflects their history as smaller number of founders was used for each of those two strains compared with the SJR strain. However, this polymorphism estimate is also downward biased by having smaller representation of fish from those two strains in the SNP discovery panel. To identify candidate SNPs for markers that can be used to distinguish between NA and European salmon we looked for SNPs that were homozygous for the reference allele in the European group and for the alternative allele in the NA group (i.e. Fst = 1). We identified 3,574 high confidence SNPs that were shared by all three bioinformatic pipelines with Fst = 1 in a comparison of genotypes from the 80 NA and 30 European Atlantic salmon. In addition, we identified 5,822 candidate SNPs with Fst = 1 from at least one of the three SNP discovery pipelines. Information on those SNPs, including the genotype of each sub-species group and the Fst value for each pipeline can be found in **Supplemental File S1**.

**Table 1:** Number and percent of polymorphic SNPs per aquaculture strain and per year-class (YC) within each strain.

Population^*^	No. of Fish^†^	No. of SNP^‡^	No. of Polymorphic	% Polymorphic	Average MAF^#^	Average Het^@^
GNB	16	6,633,346	3,595,278	0.54	0.26	0.47
GNB_YC12-13	12	6,639,956	2,775,243	0.42	0.25	0.47
GNB_YC15-16	4	6,634,773	3,061,632	0.46	0.26	0.45
PR	11	6,635,711	3,564,671	0.54	0.21	0.35
PR_YC12-13	6	6,638,954	3,132,747	0.47	0.23	0.38
PR_YC13-14	5	6,638,140	2,589,577	0.39	0.25	0.43
SJR	53	6,607,395	6,290,844	0.95	0.15	0.23
SJR_YC13-14	14	6,634,143	4,784,313	0.72	0.18	0.29
SJR_YC14-15	14	6,635,378	4,800,165	0.72	0.18	0.29
SJR_YC15-16	12	6,626,086	4,961,181	0.75	0.20	0.33
SJR_YC16-17	13	6,635,937	4,613,355	0.70	0.18	0.29

*GNB, Gaspe of New Brunswick; PR, Penobscot River; SJR, St. John River.

^†^Number of fish that were genotyped from that population or group.

^‡^Number of SNPs with genotype data for all the fish from that population or group.

^#^Average minor allele frequency (MAF) from all the polymorphic SNPs in each population.

^@^Average observed heterozygosity from all the polymorphic SNPs in each population.

## Materials and Methods

Genomic DNA was extracted from the fin clips of 80 NA Atlantic salmon selected from three aquaculture stocks that are currently propagated at the NCWMAC. These three aquaculture stocks represent three distinctive strains of NA Atlantic salmon. The GNB strain is a landlocked strain of fish. The SJR strain is of great economic importance as the fish produced by the Aquaculture industry in Northeast US and Canada were originated from this strain and it is also the primary species used in the selective breeding program in NCWMAC. The PR strain was originated from an endangered NA Atlantic salmon population. Of the 80 selected fish for DNA extraction, 16 are from GNB, 53 are from SJR, and 11 are from PR. Whole genome shotgun sequencing was performed using Illumina NovaSeq (paired-end 2 x 150 nucleotides) providing an average of 18.5x genome coverage per sample (minimum 13.7x and maximum 28x). The library preps and DNA sequencing were performed by a commercial sequencing service vendor (Admera Health, South Plainfield, NJ, USA). The information on each sample including population affiliation, number of sequence-reads, and genome sequence coverage is found in **Supplemental File S2**. All the raw sequence reads data were deposited in NCBI SRA with the BioProject accession PRJNA559280. For quality control and comparison between the NA and European Atlantic salmon, sequences of 4 DH and 31 wild European Atlantic salmon, representing a wide geographic distribution, were downloaded from NCBI SRA with the BioProject accessions PRJEB24419 (Kijas et al., 2018) and PRJEB10744 (Barson et al., 2015), respectively. The genome sequence coverage of these samples was between 4.2x and 24x.

All raw sequence reads were mapped to the Atlantic salmon genome assembly (GCF_000233375.1), using BWA mem with the default parameters (Li, 2013). At least 99.5% of the reads from each sample were mapped to the reference genome. Read pairs that are likely to have originated from duplicates of the same original DNA fragments (PCR duplicates) were marked using the Picard tools. Three pipelines, freebayes (Garrison and Marth, 2012), mpileup (Li, 2011), and GATK (Mckenna et al., 2010), were used in the variant calling step. For freebayes and mpileup pipelines, we required a minimum mapping quality score of 30 and a minimum base quality score of 20. For the GATK pipeline, read base quality scores were first recalibrated using the GATK tools and a set of high confidence SNPs were selected from the SNPs found by both freeebayes and mpileup with MAF > 0.25. Variants were then called following exactly the workflow described in the GATK Best Practice Workflow for Germline short variant discovery (Depristo et al., 2011; Van Der Auwera et al., 2013). After the variant calling step, SNPs were selected based on the following filtering steps: First, we only extracted the SNPs that are bi-allelic, not located within 5 bases distance to an indel, and have the phred-scaled variant quality score, QUAL, larger than 30; Second, We removed the SNPs that are in the low-complexity regions in the genome that were identified by RepeatMasker (http://www.repeatmasker.org/); Third, we removed the sites that were covered by more than 3,000 reads as these sites are very likely to have excess number of reads caused by repeat sequences or genome locus duplications; Fourth, we required no more than one sample with missing genotype per SNP. To call a SNP genotype for an individual sample, we required at least two reads to support calling the alternate allele and at least one read to support the reference allele; Finally, we filtered out SNP sites with heterozygous genotypes in any of the four DH lines. A workflow chart of the bioinformatic pipeline we used for SNP is found in **Supplemental File S3**.

To find the SNPs that allow for identifying the continental of origin of the Atlantic salmon, Fst between the NA and European Atlantic salmon were calculated at each selected SNP sites using the program vcftools (Danecek et al., 2011), and those with Fst = 1 were found to have different homozygous genotypes in the two groups and thus were selected as the candidate SNPs.

The SNPhylo pipeline (Lee et al., 2014) was used for generating the phylogenetic tree constructed with the SNP called by all three pipelines using the program default thresholds. The SNPs used in the analysis were required to be in the 29 chromosome sequences of the European Atlantic salmon refence genome (Lien et al., 2016) with MAF > 0.1. A linkage disequilibrium (LD) threshold of r^2^ > 0.1 was used to reduce SNP redundancy. The tree was built with the maximum likelihood method from the DNAML program within the PHYLIP package. A larger view of the phylogenetic tree is provided in **Supplemental File S4**.

## Data Availability Statement

The datasets generated for this study can be found in the DNA sequences are in NCBI SRA accession PRJNA559280. The three VCF files for the database of all the SNPs identified in this study are available online in the European Variation Archive (EVA) under project PRJEB34225 with Accessions ERZ107004, ERZ107005 and ERZ107006.

## Ethics Statement

The fish were sampled according to our Standard Operating Procedures: Care and Use of Research Animals. Publication 4, November 2018, USDA, ARS National Cold Water Marine Aquaculture Center, 25 Salmon Farm Road, Franklin, ME 04634. Approved by the Institutional Animal Care and Use Committee on November 14, 2018.

## Author Contributions

GG planned, designed, and performed the bioinformatics and data analyses and wrote the manuscript draft. CR, BP, and YP conceived the study and research plan. MP, GB, and BP designed the samples collection and collected the samples. YP, MP, and BP coordinated and co-directed the work. YP also contributed to the data analyses design and the manuscript draft. All authors reviewed the manuscript draft and approved its content.

## Funding

This study was supported by the USDA Agricultural Research Service in-house projects 8030-31000-004 and 8082-31000-012.

## Conflict of Interest

The authors declare that the research was conducted in the absence of any commercial or financial relationships that could be construed as a potential conflict of interest.
