# Supplementary material for: A New Single Nucleotide Polymorphism Database for North American Atlantic Salmon Generated Through Whole Genome Resequencing
Source: Front Genet. 2020 Feb 21;11:85. doi: 10.3389/fgene.2020.00085 (PMC7046687; doi:10.3389/fgene.2020.00085)
Supplement: Supplementary file 1 [file DataSheet_1.pdf]

Supplementary File S3. A workflow chart of the bioinformatic pipeline we used for SNP discovery from whole-genome resequencing.

- ❑ SNP calling pipeline

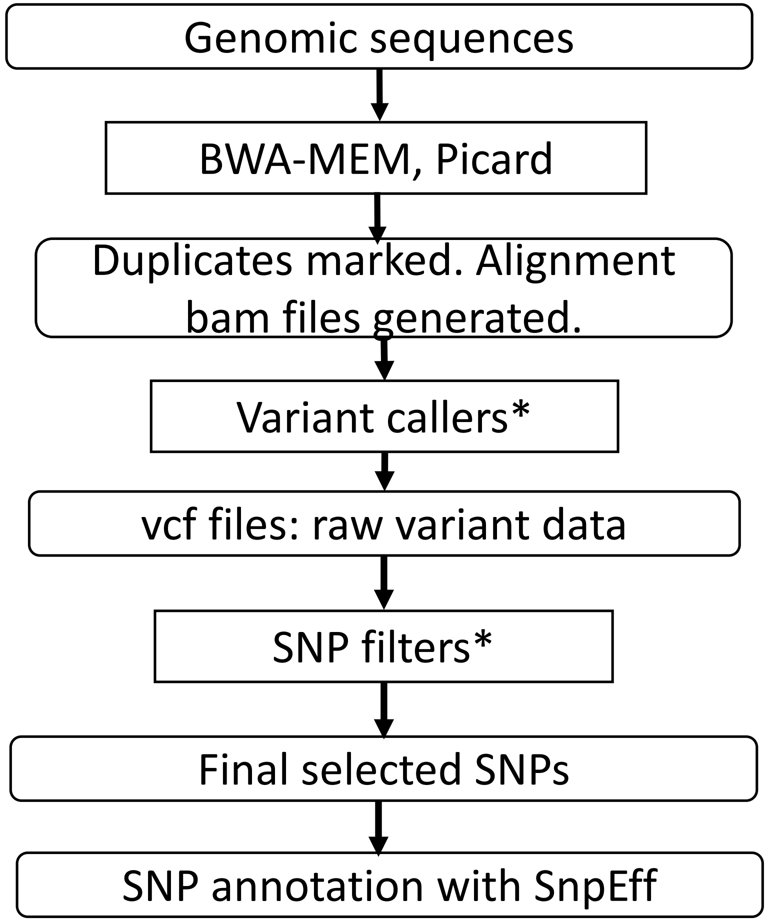

- ❑ Variant callers: freebayes, samtools mpileup, and GATK

- ❑ SNP filters

**QUAL filter**	Required variant calling quality score QUAL > 30
**NS filter**	Required no more than one sample with a missing genotype per SNP
**DP filter**	Required reads depth <3,000 per SNP
**Repeat filter**	Required that the SNPs were not in the sites marked as “Simple repeat” or “Low complexity” by RepeatMasker
**DH filter**	Required no heterozygous genotype for any of the four double-haploid fish
