# Supplementary material for: A New Single Nucleotide Polymorphism Database for North American Atlantic Salmon Generated Through Whole Genome Resequencing
Source: Front Genet. 2020 Feb 21;11:85. doi: 10.3389/fgene.2020.00085 (PMC7046687; doi:10.3389/fgene.2020.00085)

Supplementary File S4.
A phylogenetic tree of the 80 North American Atlantic salmon sampled from three aquaculture strains.

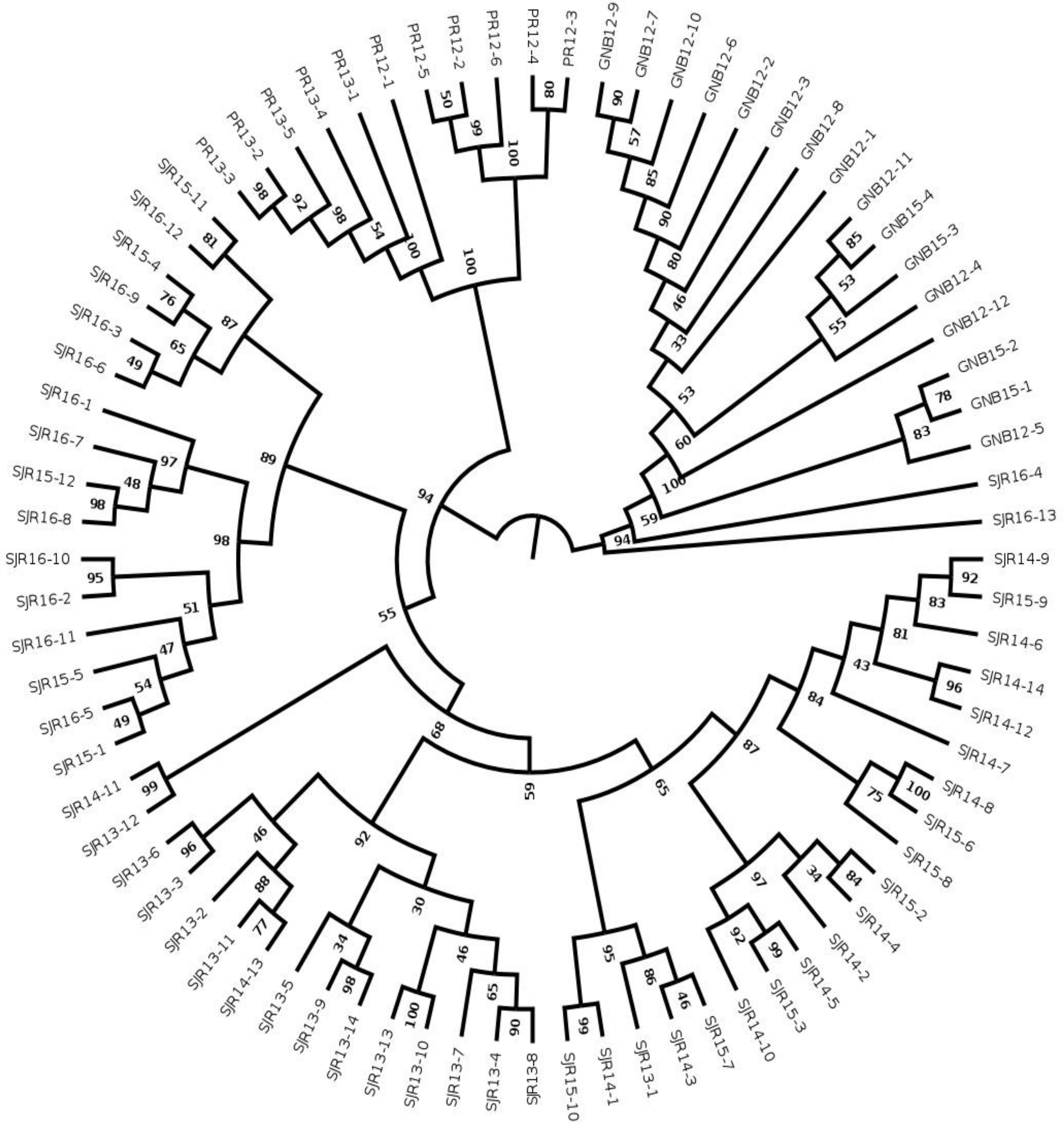

Supplement: Supplementary file 2 [file DataSheet_2.pdf]
